# Supplementary material for: The FDA-Approved Drug Pyrvinium Selectively Targets ER+ Breast Cancer Cells with High INPP4B Expression
Source: Cancers (Basel). 2022 Dec 26;15(1):135. doi: 10.3390/cancers15010135 (PMC9817693; doi:10.3390/cancers15010135)
Supplement: Supplementary file 1 [file cancers-15-00135-s001.zip › cancers-2025205_Supplementary_Materials.docx.pdf]

Supplementary Materials

The FDA-approved drug pyrvinium selectively targets ER⁺ breast cancer cells with high INPP4B expression

Samuel J. Rodgers, Lisa M. Ooms and Christina A. Mitchell

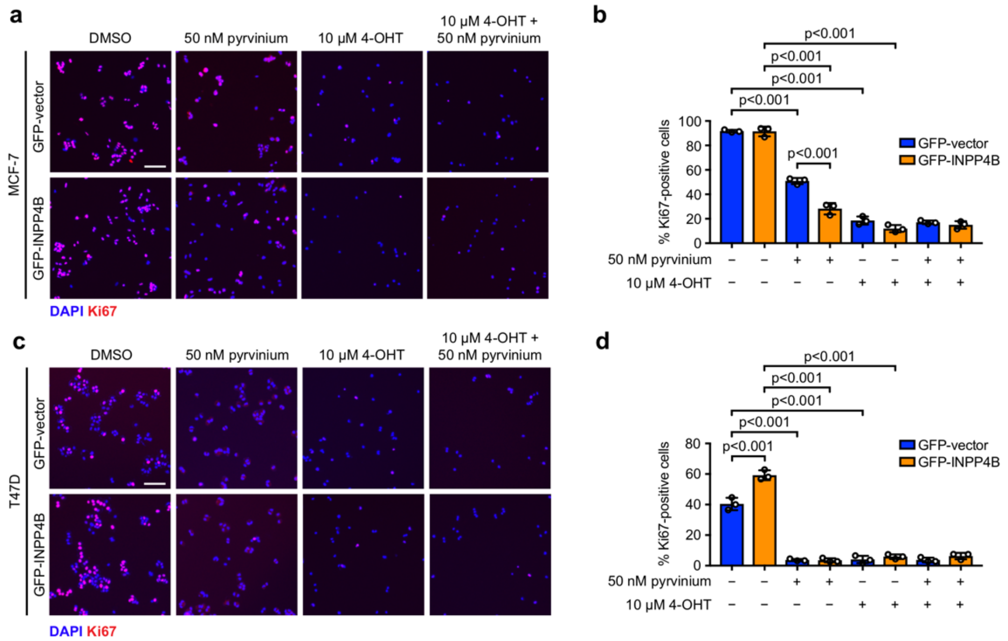

Figure S1. Pyrvinium or 4-OHT treatment suppress the proliferation of ER⁺ breast cancer cells. MCF-7 (**a**, **b**) or T47D (**c**, **d**) cells expressing GFP-vector or GFP-INPP4B were treated with 50 nM pyrvinium and/or 10 μM 4-OHT or DMSO as a vehicle control for 48 *h*. Cells were fixed and immunostained with Ki67 antibodies, and co-stained with DAPI (**a**, **c**). Data represent the percentage of Ki67-positive cells ± SD (n=3 experiments, >200 cells/experiment) (**b**, **d**). Scale bar is 100 μm in **a**, **c**. + indicates where treatment was added, and – indicates where no treatment was added. *p* values were determined by one-way ANOVA with Tukey post-hoc test in **b**, **d**.

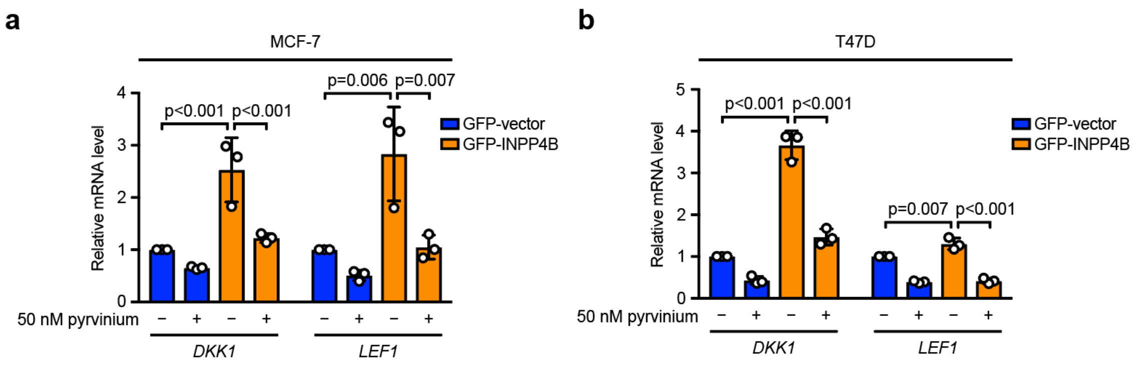

Figure S2. Pyrvinium suppresses Wnt target gene expression in INPP4B-overexpressing ER$^+$ breast cancer cells. MCF-7 (**a**) or T47D (**b**) cells expressing GFP-vector or GFP-INPP4B were treated with 50 nM pyrvinium or DMSO as a vehicle control for 24 *h*. RNA was extracted and two-step quantitative RT-PCR was performed using primers for *LEF1* or *DKK1*, and normalized to *RRN18S*. Expression was determined using the ΔΔCt method and expressed relative to DMSO-treated GFP-vector control cells (± SD), which were assigned an arbitrary value of 1 (n = 3 experiments). + indicates where treatment was added, and – indicates where no treatment was added. *p* values were determined by one-way ANOVA with Tukey post-hoc test in **a**, **b**.
